# Dietary Sources of Nutrient Consumption in a Rural Japanese Population

**DOI:** 10.2188/jea.12.1

**Published:** 2007-11-30

**Authors:** Keiko Ogawa, Yoshitaka Tsubono, Yoshikazu Nishino, Yoko Watanabe, Takayoshi Ohkubo, Takao Watanabe, Haruo Nakatsuka, Nobuko Takahashi, Mieko Kawamura, Ichiro Tsuji, Shigeru Hisamichi

**Affiliations:** 1 Department of Public Health, Tohoku University Graduate School of Medicine, Miyagi, Japan.; 2 Department of Environmental Health, Miyagi University of Education.; 3 School of Nursing, Miyagi University.; 4 Faculty of Human Sciences, Sendai Shirayuri Women’s College.; 5 Faculty of Human Life and Environmental Science, Kochi State Women’s University.

**Keywords:** Japanese diet, nutrient, dietary sources

## Abstract

We determined the sources of nutrient intake of 59 men and 60 women in two rural towns in the Miyagi Prefecture, a northeastern part of Japan. Four 3-day food records were collected in four seasons within a year. The total dishes and recipes were classified into 197 items. Their percent contributions to the total population consumption of energy and 14 nutrients were calculated as the sum of the nutrient intake contributed by a given dish or recipe divided by the total nutrient intake from all the items. Rice was the largest contributor for energy (29.8%), protein (13.0%) and carbohydrates (45.3%). Miso soup, as a dish, was a leading contributor (7.1%) for fat. The largest contributor for sodium, calcium, carotene, vitamin C were miso soup (17.1%), milk (16.6%), spinach (23.6%), green tea (13.6%), respectively. The result suggests that the examination of nutrient sources based on dishes and recipes, rather than on food materials, may be useful in characterizing the dietary patterns of populations.

## INTRODUCTION

Public health workers often give nutritional education in terms of nutrient contents of dishes and recipes. Therefore examining the contribution of individual dishes and recipes, rather than food materials, is important because the dishes and recipes are the unit of eating.

Several studies have reported dietary sources of nutrient consumption among populations in the US^1^^-^^4^^)^, Italy^5^^,^^6^^)^, UK^7^^)^, Germany^8^^)^ and Japan^9^^-^^12^^)^. Some studies examined or compared dietary sources of different groups of general population^13^^-^^18^^)^. However, most studies examined the contribution of foods or food materials, rather than dishes and recipes.

In this study, based on diet records collected from 119 subjects in rural Japan, we classified all the dishes and recipes into 197 items to study the percent contribution for the total population intake of 15 nutrient variables. We also compared our results with studies conducted in western and the Japanese populations.

## SUBJECTS AND METHODS

### Study design and subjects

The subjects were residents in two rural towns in Miyagi prefecture in the northern part of the mainland Japan, who were a subsample of participants in the two cohort studies that were started in 1990^19^^)^ and 1994^20^^)^. Fifty-nine men (mean age=62.5±8.4SD) and 60 women (mean age=61.2±8.5SD) were selected on the voluntary basis. Written informed consent was obtained from all the subjects.

### Dietary records

Diet records were collected on three consecutive days four times in a year during November, 1996 through November, 1997. The records were collected in a way to cover both weekdays and weekends by avoiding the same days of the week, and also to cover four different seasons of the year (November, 1996; February, 1997; May, 1996; August, 1997). In total, 113 participants provided all the 12-day food records, 9-day records from 4 participants, 6-day records from 1 participant and 3-day records from 1 participant. The participants were instructed to record all foods and beverages consumed in a standardized booklet. They were asked to provide detailed descriptions of each food (open-ended) including the weights prepared and proportions consumed. The participants recorded their diets by themselves and research dietitians checked their records in a standardized way after the completion by the participants. The daily consumption of 15 nutrient variables was calculated from these records using Standard Tables of Food Composition published by the Science and Technology Agency of Japan^21^^)^.

### Dish/recipe lists

Research nutritionists classified all the dish and recipes into 197 categories by considering the similarities in terms of nutrient contents and perceptions of subjects. First, we numbered all the foods by using a published book^22^^)^ (which classifies all the dishes) as a sample. Cooking oils and soy sauce with specific dishes or recipes were included into those dish/recipe categories. For example, soy sauce could be included in “roast fish” and “sashimi”, or cooking-oil was included in “fries with vegetables”, “fried eggs” and “tempra” etc. Miso soup was used as a recipe category, which included miso (bean paste), vegetables and several protein foods such as eggs. We assigned a single category for all types of miso soup.

### Calculations of contribution rates of dish/recipe items for the total population intake of nutrients

The percent contribution of each dish or recipe to the total population intake of energy and 14 nutrient variables was computed according to the procedures described by Block et al^23^^)^. Fourteen nutrient variables presented were as follows: protein, fat, carbohydrates, calcium, sodium, potassium, phosphorus, iron, retinol, carotene, vitamin C, vitamin B1, vitamin B2, niacin. The 197 dishes/recipes were ranked according to their percent contributions to the total consumption of a nutrient.

There were no marked differences between men and women regarding the percentage contributions of dish/recipe items to the total nutrient intakes. Therefore we presented the results for men and women combined.

## RESULTS

The participants were aged 45-77 y in men and 47-76 in women. Their major occupations were farmers, self-employed and house-wives. The percentage of current smokers in men was 49.6% (28 subjects). No women currently smoked. Mean daily intake of both men and women were presented elsewhere^24^^)^.

Table 1 presents the percentage contributions of dish/recipe items to the total intake of energy and macronutrients (protein, fat, carbohydrates). Rice was the largest source of energy, contributing 29.8% of total intake, followed by miso soup (4.8%). The largest contributor for protein was rice (13.0%), followed by miso soup (8.7%). Rice was the largest contributor (45.3%) for carbohydrates, followed by miso soup (3.4%). Miso soup was a leading contributor (7.1%) for fat, followed by milk (6.7%). In comparison with large contribution of the highest ranked item in energy (rice, 29.8%) and carbohydrates (rice, 45.3%), there was no remarkable single contributor for fat. When rice and miso soup, which were consumed frequently among a rural Japanese (78.2 per 100 person-meals for rice and 53.5 for miso soup), were combined, they were the most important contributors of macronutrients; 34.6% of total for energy, 21.7% for protein, 48.7% for carbohydrates and 11.2% for fat.

**Table 1.** Percentage contribution of dish/recipe items to the total intake of energy and macronutrients (protein, carbohydrates and fat).

	Energy			Protein		

Rank	Description	% to the total	Cumulative %	Description	% to the total	Cumulative %
1	Rice	29.8	29.8	Rice	13.0	13.0
2	Miso soup	4.8	34.6	Miso soup	8.7	21.7
3	milk	3.2	37.8	Roast fish	7.5	29.3
4	Japanese noodle	2.7	40.4	Raw fish	6.2	35.5
5	Roast fish	2.6	43.0	milk	3.8	39.3
6	Sake	2.4	45.4	Fermented soy bean	3.3	42.5
7	Japanese sweets	2.2	47.6	Fish boiled with soy	2.8	45.3
8	Curry and rice	1.8	49.4	Japanese noodle	2.4	47.7
9	Fermented soybean	1.6	51.0	Vegetables boiled with soy	2.2	49.9
10	Beer	1.6	52.6	Sushi	1.6	51.5
11	Raw fish	1.6	54.2	Spinach	1.5	53.0
12	Vegetables boiled with soy	1.5	55.7	Fried egg	1.5	54.5
13	Sushi	1.4	57.1	Fries with vegetables	1.4	56.0
14	Fries with vegetables	1.4	58.5	Dish with tofu	1.3	57.3
15	Chinese noodle	1.2	59.6	Grilled meat	1.3	58.6
16	Fried egg	1.0	60.7	Fried fish	1.1	59.7
17	Grilled meat	1.0	61.7	Raw egg	1.1	60.9
18	Fish boiled with soy	1.0	62.7	Chinese noodle	1.1	62.0
19	Tempra	1.0	63.7	Eggroll	1.1	63.1
20	Breads	1.0	64.6	Dried fish	1.1	64.2
21	Rice with assorted mixtures	1.0	65.5	Curry and rice	1.0	65.2
22	Rice cake	0.9	66.4	Boiled fish paste	1.0	66.2
23	Eggroll	0.9	67.3	Japanese sweets	1.0	67.2
24	Cake	0.8	68.1	Fried chicken	1.0	68.2
25	Rice cracker	0.8	68.8	Food cooked in the pot	1.0	69.1
26	Precooked Chinese noodle	0.8	69.6	Cuttlefish	1.0	70.0
27	Dish with tofu	0.7	70.3	Dish with eggs	0.8	70.8
28	Fried pork cutlet	0.7	71.0	Tempra	0.8	71.6
29	Fried chicken	0.7	71.7	Fried pork cutlet	0.7	72.3
30	Liquor (Shouchu)	0.7	72.4	Buckwheat noodle	0.7	73.0

	Carbohydrates			Fat		

1	Rice	45.3	45.3	Miso soup	7.1	7.1
2	Miso soup	3.4	48.7	Milk	6.7	13.8
3	Japanese sweets	3.3	52.0	Roast fish	4.9	18.7
4	Japanese noodle	3.1	55.0	Rice	4.2	22.8
5	Curry and rice	1.8	56.9	Fries with vegetables	3.5	26.3
6	Milk	1.8	58.6	Fermented soy bean	3.1	29.4
7	Sushi	1.6	60.2	Fried egg	3.0	32.4
8	Chinese noodle	1.4	61.5	Grilled meat	3.0	35.4
9	Vegetables boiled with soy	1.3	62.8	Vegetables boiled with soy	2.6	38.0
10	Rice with assorted mixtures	1.2	64.0	Curry and rice	2.5	40.5
11	Rice cake	1.2	65.3	Eggroll	2.4	42.9
12	Breads	1.1	66.3	Tempra	2.0	44.8
13	Rice cracker	1.0	67.3	Fried pork cutlet	1.9	46.7
14	Pumpkins boiled with soy	1.0	68.3	Japanese noodle	1.9	48.6
15	Apple	0.9	69.2	Fried chicken	1.8	50.4
16	Persimmon	0.9	70.1	Dish with tofu	1.7	52.1
17	Mandarin orange	0.9	71.1	Raw fish	1.6	53.8
18	Beer	0.9	71.9	Raw egg	1.6	55.3
19	Coffee drink,canned	0.8	72.7	Fish fried with soy	1.5	56.8
20	Cake	0.8	73.5	Fried fish	1.4	58.3
21	Buckwheat noodle	0.8	74.3	Dish with eggs	1.3	59.6
22	Sake	0.8	75.1	Cake	1.3	60.9
23	Yogurt	0.8	75.9	Precooked Chinese noodle	1.2	62.0
24	Fermented soy bean	0.8	76.6	Potato salad	1.1	63.1
25	Chinese fried rice	0.7	77.3	Ham, sausage	1.1	64.2
26	Precooked Chinese noodle	0.7	78.0	Peanut	1.1	65.2
27	Tempra	0.7	78.7	Breads	1.0	66.3
28	Coffee	0.6	79.3	Fresh vegetable salad	1.0	67.2
29	Fries with vegetables	0.6	79.9	Sushi	1.0	68.2
30	Bananas	0.6	80.5	Biscuit	1.0	69.2

Table 2a and 2b present the percentage contribution of dish/recipe items to the total intake of minerals (sodium, potassium, calcium, phosphorus and iron). Miso soup was a major contributor for minerals; ranked first for sodium (17.1%) and potassium (10.6%) and second for calcium (16.6%). Milk was the largest contributor (16.6%) of calcium.

**Table 2a.** Percentage contribution of dish/recipe items to the total intake of sodium, potassium, and calcium.

	Sodium			Potassium			Calcium		

Rank	Description	% to the total	Cumulative %	Description	% to the total	Cumulative %	Description	% to the total	Cumulative %
1	Miso soup	17.1	17.1	Miso soup	10.6	10.6	Milk	16.6	16.6
2	Roast fish	7.3	24.5	Milk	5.5	16.0	Miso soup	16.3	32.9
3	Japanese noodle	3.9	28.3	Rice	4.0	20.1	Spinach	4.4	37.3
4	Pickled cucumber	3.6	31.9	Spinach	3.8	23.8	Vegetables boiled with soy	3.5	40.8
5	Vegetables boiled with soy	3.2	35.1	Vegetables boiled with soy	3.7	27.5	Yogurt	2.7	43.5
6	Pickled radish	2.9	38.0	Roast fish	3.5	31.0	Dish with tofu	2.6	46.1
7	Fermented soybean	2.8	40.8	Fermented soybean	3.5	34.4	Low fat milk	2.4	48.6
8	Pickled plum	2.8	43.6	Raw fish	3.3	37.7	Roast fish	2.4	50.9
9	Raw fish	2.3	45.9	Green tea	2.6	40.4	Fermented soybean	2.2	53.1
10	Spinach	2.1	48.0	Fries with vegetables	2.2	42.5	Dried fish	1.9	55.0
11	Fries with vegetables	2.0	50.0	Pickled cucumber	1.6	44.1	Rice	1.6	56.7
12	Fish boiled with soy	1.7	51.8	Fish boiled with soy	1.6	45.6	Green tea	1.3	58.0
13	Sushi	1.4	53.1	Tomato	1.4	47.0	Fries with vegetables	1.3	59.3
14	Chinese noodle	1.3	54.5	Pumpkins boiled with soy	1.3	48.3	Japanese noodle	1.2	60.5
15	Pickled vegetables	1.3	55.8	Seaweeds	1.3	49.6	Hijiki(seaweeds)	1.2	61.7
16	Boiled tish paste	1.2	57.0	Curry and rice	1.3	50.9	Coffee drink,canned	1.1	62.8
17	Precooked Chinese noodle	1.2	58.2	Coffee	1.2	52.1	Pickled cucumber	1.0	63.8
18	Rice	1.2	59.5	Mandarin orange	1.2	53.2	Food cooked in the pot	0.9	64.7
19	Curry and rice	1.2	60.6	Japanese noodle	1.2	54.4	Wild plant	0.9	65.6
20	Salmon roe,ikra,sea urchin	1.0	61.6	Chinese noodle	1.1	55.5	Fried egg	0.9	66.5
21	Pickled eggplant	1.0	62.7	Persimmon	1.0	56.5	Tempra	0.8	67.3
22	Wild plant	1.0	63.7	Bananas	1.0	57.4	Dish with eggs	0.8	68.1
23	Meat chouder with vegetables	1.0	64.6	Wild plant	0.9	58.4	Cheese	0.8	68.9
24	Fried egg	0.9	65.6	Beer	0.9	59.3	Mandarin orange	0.8	69.6
25	Milk	0.9	66.5	Fresh vegetable salad	0.9	60.2	Eggroll	0.7	70.3
26	Seaweeds	0.9	67.4	Food cooked in the pot	0.9	61.1	Seaweeds	0.7	71.1
27	Dried fish	0.9	68.3	Tempra	0.9	62.0	Grated radish	0.7	71.8
28	Dish with tofu	0.9	69.2	Yarm	0.9	62.9	Japanese sweets	0.7	72.4
29	Pickled Chinese cabbage	0.8	70.0	Dish with eggs	0.8	63.7	Raw fish	0.7	73.1
30	Food boiled down with soy	0.8	70.8	Grilled meat	0.8	64.6	Meat chouder with vegetables	0.7	73.8

**Table 2b.** Percentage contribution of dish/recipe items to the total intake of phosphorus and iron.

	Phosphorus			Iron			

Rank	Description	% to the total	Cumulative %	Description	% to the total	Cumulative %
1	Rice	10.9	10.9	Miso soup	16.2	16.2
2	Miso soup	9.6	20.5	Boiled greens with soy sauce	5.3	21.5
3	Milk	8.3	28.8	Fermented soybeam	4.5	26.0
4	Roast fish	5.7	34.4	Rice	3.9	29.9
5	Sashimi (raw fish)	4.6	39.0	Green tea	3.6	33.5
6	Fermented soybeam	2.8	41.8	Sashimi (raw fish)	3.4	36.9
7	Vegetables boiled hard with soy	2.2	44.0	Vegetables boiled hard with soy	3.4	40.3
8	Fish broiled with soy	2.1	46.1	Roast fish	3.0	43.3
9	Japanese noodle (Udon)	1.8	47.9	Japanese noodle (Udon)	2.0	45.3
10	Boiled greens with soy sauce	1.8	49.7	Dish with bean curd (tofu)	1.9	47.3
11	Fried egg	1.8	51.4	Seaweeds(hijiki)	1.9	49.2
12	Dried fish	1.4	52.9	Fries with meat and vegetables	1.8	51.0
13	Sushi(vinegard fish and rice)	1.4	54.3	Fried egg	1.6	52.6
14	Fries with meat and vegetables	1.4	55.6	Japanese style sweets	1.3	53.9
15	Raw egg	1.3	56.9	Shellfish	1.3	55.2
16	Dish with eggs	1.3	58.2	Fish broiled with soy	1.3	56.5
17	Yogurt	1.3	59.5	Chinese noodle	1.2	57.7
18	Dish with bean curd (tofu)	1.2	60.7	Raw egg	1.2	59.0
19	Fried fish	1.0	61.7	Eggroll	1.2	60.1
20	Beer	1.0	62.7	Sushi(vinegard fish and rice)	1.1	61.2
21	Curry and rice	1.0	63.6	Curry and rice	1.1	62.3
22	Low fat milk	0.9	64.6	Grilled meat	1.0	63.4
23	Dish with eggs	0.9	65.5	Milk	1.0	64.4
24	Grilled meat	0.8	66.3	Dish with eggs	1.0	65.4
25	Chinese noodle	0.8	67.1	Food cooked in the pot	0.9	66.3
26	Food cooked in the pot	0.8	68.0	Wild plant	0.9	67.2
27	Tempra (Japanese deep-fat fried fish)	0.8	68.8	Food boiled down in soy	0.8	68.0
28	Japanese noodle (Soba): buckwheat noodle	0.7	69.5	Meat chouder with assorted vegetables	0.8	68.7
29	Cuttlefish, squid	0.7	70.2	Pickled cabbage and cucumber	0.7	69.5
30	Japanese style sweets	0.7	71.0	Tempra (Japanese deep-fat fried fish)	0.7	70.2

Table 3a and 3b present the percentage contribution of dish/recipe items to the total intake of vitamins (carotene, retinol, vitamin C, vitamin B1, vitamin B2, and niacin). The largest contributor for carotene was spinach (23.6%), followed by miso soup (10.4%). Pork liver and chicken liver were major contributors, constituting of 23.4 % of total retinol intake although they were seldom consumed (0.26 for pork 0.19 for chicken per 100 person-meals). In case of vitamin C, green tea (consumed 48.3 per 100 person-meals) was the largest contributor (13.6%), followed by spinach (9.0%) and miso soup (8.6%).

**Table 3a.** Percentage contribution of dish/recipe items to the total intake of carotene, retinol, and vitamin C.

	Carotene			Retinol			Vitamin C		

Rank	Description	% to the total	Cumulative %	Description	% to the total	Cumulative %	Description	% to the total	Cumulative %
1	Spinach	23.6	23.6	Liver, pork	14.4	14.4	Green tea	13.6	13.6
2	Miso soup	10.4	33.9	Milk	9.1	23.5	Spinach	9.0	22.5
3	Vegetables boiled with soy	8.5	42.4	Liver, chicken	9.0	32.5	Miso soup	8.6	31.1
4	Fries with vegetables	4.3	46.7	Fried egg	5.0	37.5	Mandarin orange	6.5	37.6
5	Dish with eggs	3.2	49.9	Fish split grilled in soy	4.8	42.3	Persimmon	5.5	43.1
6	Curry and rice	3.1	53.0	Grilled meat	4.8	47.1	Fries with vegetables	3.8	46.9
7	Pumpkins boiled with soy	2.8	55.8	Raw egg	4.4	51.5	Orange	3.4	50.3
8	Vegetable juice	2.4	58.2	Eggroll	4.4	55.8	Tomato	2.8	53.1
9	Wild plant	2.3	60.5	Miso soup	4.2	60.0	Pumpkins boiled with soy	2.7	55.8
10	Tomato	2.2	62.6	Fish boiled with soy	4.2	64.2	Pickled cucumber	2.5	58.3
11	Chopped burdock root with soy	1.8	64.4	Roast fish	3.8	68.0	Vegetables boiled with soy	2.4	60.6
12	Hijiki(seaweeds)	1.7	66.1	Rice with grilled eel	3.0	71.0	Strawberry	1.9	62.5
13	Pickled cucumber	1.6	67.7	Dish with eggs	2.1	73.1	Boiled vegetables	1.9	64.4
14	Fresh vegetable salad	1.5	69.2	Raw fish	1.7	74.7	Fresh vegetable salad	1.9	66.3
15	Toasted laver	1.4	70.6	Hard boiled egg	1.5	76.3	Pickled Chinese cucumber	1.6	67.9
16	Stewed vegetables with milk	1.3	72.0	Breads	1.2	77.5	Melon	1.3	69.2
17	Potato salad	1.3	73.3	Japanese noodle	1.2	78.7	Vegetable juice	1.3	70.5
18	Watermelon	1.3	74.6	Cake	1.1	79.8	Dish with eggs	1.1	71.5
19	Boiled vegetables	1.1	75.7	Fried fish	1.0	80.8	Fried egg	1.0	72.6
20	Pork chouder	1.0	76.7	Fermented soybean	1.0	81.8	Tempra	0.9	73.5
21	Sushi	0.9	77.6	Sushi	1.0	82.7	100% fresh juice	0.9	74.4
22	Japanese noodle	0.9	78.6	Fries with vegetables	0.9	83.6	Kiwi fruit	0.9	75.3
23	Mandarin orange	0.8	79.4	Salted fish guts	0.9	84.5	Curry and rice	0.8	76.1
24	Tempra	0.8	80.2	Vegetables boiled with soy	0.9	85.4	Grilled meat	0.8	76.9
25	Oden	0.8	81.0	Pot-steamed hotchpotch	0.8	86.2	Grated radish	0.8	77.6
26	Food cooked in the pot	0.8	81.8	Chicken boiled with soy	0.8	87.0	Potato salad	0.7	78.3
27	Vinegard vegetables	0.8	82.6	Salmon roe,ikra,sea urchin	0.7	87.7	Pickled radish	0.7	79.0
28	Pickled greens	0.8	83.3	Dried fish	0.7	88.3	Other drinks(pokari)	0.7	79.7
29	Rice with assorted vegetables	0.7	84.0	Fried chickens	0.6	88.9	Food cooked in the pot	0.7	80.3
30	Rice	0.7	84.8	Cheese	0.6	89.5	Pickled greens	0.6	81.0

**Table 3b.** Percentage contribution of dish/recipe items to the total intake of vitamin B1, vitamin B2 and niacin.

	Vitamin B1			Vitamin B2			Niacin		

Rank	Description	% to the total	Cumulative %	Description	% to the total	Cumulative %	Description	% to the total	Cumulative %
1	Rice	12.9	12.9	Milk	10.6	10.6	Sashimi (raw fish)	13.0	13.0
2	Miso soup	8.0	20.9	Green tea	8.5	19.1	Roast fish	9.8	22.7
3	Roast fish	3.5	24.4	Miso soup	6.1	25.1	Rice	7.6	30.3
4	Fries with meat and vegetables	3.5	27.9	Fermented soybeam	5.8	30.9	Miso soup	7.3	37.6
5	Milk	3.2	31.1	Roast fish	4.5	35.4	Fish broiled with soy	3.6	41.2
6	Grilled meat	3.1	34.3	Rice	3.1	38.5	Beer	2.8	44.0
7	Sashimi (raw fish)	2.9	37.1	Boiled greens with soy sauce	2.9	41.4	Green tea	2.5	46.6
8	Japanese noodle (Udon)	2.7	39.8	Fried egg	2.8	44.2	Vegetables boiled hard with soy	2.1	48.7
9	Boiled greens with soy sauce	2.4	42.2	Raw egg	2.4	46.6	Fries with meat and vegetables	2.1	50.8
10	Fried pork cutlet	2.3	44.6	Eggroll	2.3	48.9	Grilled meat	1.8	52.6
11	Curry and rice	2.3	46.9	Fish broiled with soy	2.0	50.9	Japanese noodle (Udon)	1.6	54.3
12	Mandarin orange	2.2	49.1	Sashimi (raw fish)	2.0	52.9	Boiled greens with soy sauce	1.6	55.9
13	Vegetables boiled hard with soy	2.1	51.2	Vegetables boiled hard with soy	2.0	54.9	Coffee	1.6	57.5
14	Fish broiled with soy	1.7	52.9	Yogurt	1.6	56.6	Sushi(vinegard fish and rice)	1.6	59.1
15	Fried egg	1.4	54.3	Beer	1.6	58.1	Fried fish	1.3	60.4
16	Dish with bean curd (tofu)	1.4	55.7	Fries with meat and vegetables	1.6	59.7	Curry and rice	1.3	61.7
17	Fermented soybeam	1.3	57.0	Japanese noodle (Udon)	1.5	61.3	Chicken fried without coat	1.3	63.0
18	Chinese noodle	1.1	58.1	Dish with eggs	1.4	62.7	Food cooked in the pot	1.2	64.1
19	Sushi(vinegard fish and rice)	1.1	59.2	Low fat milk	1.4	64.1	Fermented soybeam	1.2	65.3
20	Ham, sausage	1.0	60.2	Grilled meat	1.2	65.3	Dried fish	1.1	66.4
21	Pumpkins boiled hard with soy	1.0	61.3	Sushi(vinegard fish and rice)	0.9	66.2	Chinese noodle	1.1	67.5
22	Pork chouder	1.0	62.3	Fried fish	0.9	67.1	Peanut	1.0	68.5
23	Food cooked in the pot	1.0	63.3	Coffee drink, canned	0.8	67.9	Fried pork cutlet	1.0	69.5
24	Japanese noodle	0.9	64.2	Hard boiled egg	0.8	68.7	Cuttlefish, squid	0.8	70.3
25	Tomato	0.9	65.0	Dried fish	0.8	69.6	Ham, sausage	0.7	71.0
26	Corn	0.8	65.8	Food cooked in the pot	0.8	70.4	Cod roe	0.7	71.7
27	Fresh vegetable salad	0.8	66.6	Liver, pork	0.8	71.2	Milk	0.7	72.5
28	Tempra	0.8	67.4	Chinese noodle	0.7	71.9	Corn	0.7	73.2
29	Dish with eggs	0.7	68.1	Shellfish	0.6	72.6	Meat chouder with assorted vegetables	0.7	73.9
30	Rice	0.7	68.9	Mandarin orange	0.6	73.2	Tempra	0.7	74.5

## DISCUSSION

We presented dish/recipe based dietary sources to the total nutrient intakes. Several cautions should be required in interpreting the results. For instance, miso soup was used as a category of a recipe in this study, so that it included not only bean paste but also several vegetables and protein-rich foods such as eggs and tofu. There were not cooking oils in the list of contributors for fat intake because they were not dishes and included in other categories of dishes or recipes.

Table 4 summarizes the studies conducted in western countries and Japan regarding the dietary sources for the nutrient intakes. We compared our result with those of other studies conducted in general (adult) population. It gives us the information about the characteristics of habitual diet of their population although dietary sources depend on the method of classification. There are some differences between Japanese and western diets. Rice was the largest contributor for energy, protein and carbohydrates in Japanese populations. In western populations, breads and beef were major contributors for most of macronutrients. Milk was the largest contributor for calcium in all the populations.

**Table 4.** The comparison of the largest sources of energy and nutrient intakes in various population.

Author	Present study	Tokudome^l0^^)^ et al	Tsubono^9^^)^	Block^1^^,^^2^^)^	Subar^4^^)^	Krebs-Smith^3^^)^	Knogh^5^^,^^6^^)^ et al	Cade^7^^)^	Winkler^8^^)^
Population	Japanese	Japanese	Japanese	USA	USA	USA	Italy	UK	German

Energy	Rice	Rice	Rice	White breads	Yeast breads	Yeast breads	White bread	White bread	Sausages
	(29.8%)	(31.0%)	(35.0%)	(9.6%)	(9.8%)	(6.9%)	(15.7%)	(11.7%)	(8.8%)
Protein	Rice	Rice	Rice	Beef	Beef	Poultry	White bread	White bread	–
	(13.0%)	(14.7%)	(17.1%)	(12.6%)	(17.7%)	(12.3%)	(13.1%)	(10.5%)	
Carbohydrates	Rice	Rice	Rice	White breads	Yeast breads	Yeast breads	White bread	White bread	–
	(45.3%)	(50.5%)	(53.6%)	(15.0%)	(14.9%)	(11.1%)	(28.5%)	(20.4%)	
Fat	Miso soup	Chicken egg	Vegetable oils	Hamburgers	Beef	Salad dressings	Olive oil	Milk	Sausages
	(7.1%)	(10.3%)	(16.4%)	(7.0%)	(11.1%)	(9.2%)	(32.4%)	(9.6%)	(16.5%)
Sodium	Miso soup	–	Soy sauce	White breads	Tablet salt	–	–	–	–
	(17.1%)		(23.2%)	(12.1%)	(23.4%)				
Potassium	Miso soup	–	Rice	Coffee, tea	Milk	–	–	–	–
	(10.6%)		(8.0%)	(8.6%)	(12.5%)				
Calcium	Milk	Milk	Milk	Whole milk	Milk	–	Whole milk	Milk	–
	(16.6%)	(13.0%)	(15.8%)	(22.0%)	(34.2%)		(17.0%)	(29.1%)	
Phosphorus	Rice	–	Rice	Whole milk	Milk	–	–	–	–
	(10.9%)		(23.4%)	(10.5%)	(16.7%)				
Iron	Miso soup	Chicken egg	Rice	White breads	Cereal	–	Wine	White bread	–
	(16.2%)	(9.0%)	(8.8%)	(11.4%)	(14.6%)		(19.3%)	(14.9%)	
Carotene	Spinach	Carrot	Carrot	–	Carrot	–	–	–	Pork
	(23.6%)	(41.0%)	(48.9%)		(53.0%)				(20.9%)
Retinol	Liver, pork	–	Liver, pork	–	–	–	Liver	Offal meat	–
	(14.4%)		(38.0%)				(13.7%)	(44.2%)	
Vitamin C	Green tea	Spinach	Spinach	Orange juice	Orange	–	Orange	Potatoes	Potatoes
	(13.6%)	(10.0%)	(11.2%)	(26.50%)	(23.7%)		(19.3%)	(26.2%)	(23.4%)
Vitamin B1	Rice	–	Pork	White breads	Yeast breads	–	Pasta	–	–
	(12.9%)		(21.6%)	(17.8%)	(16.4%)		(12.3%)		
Vitamin B2	Milk	–	Egg	Whole milk	Milk	–	Whole milk	–	–
	(10.6%)		(14.7%)	(13.4%)	(18.5%)		(14.5%)		
Niacin	Sashimi	–	Rice	White breads	Poultry	–	–	–	–
	(13.0%)		(15.9%)	(9.5%)	(15.1%)				

There are some differences among the studies in Japanese populations. For example, in the present study, miso soup, as a recipe, was the largest contributor for fat. In comparison, Tokudome^10^^)^ reported chicken egg and Tsubono^9^^)^ reported vegetable oils as the largest contributor for fat. This is partly because we used miso soup as a recipe category while the other two studies considered bean paste as a single food material. In case of carotene, miso soup was the second largest contributor in our study. Carrot was ranked as the largest contributor in other studies for Japanese populations, but not ranked in our study because carrot was classified into several dishes, not as a food. The very high contribution of green tea to vitamin C intake is characteristics of the present study. The subjects of our study consumed green tea 48.3 per 100 person-meals. On the other hand, it was consumed only 16.7 per 100 person-meals in another study^9^^)^. One possible reason about the large contribution of green tea may be that the mean age of our subjects were relatively high (men: 62.5 ± 8.4 SD; women: 61.2 ± 8.5 SD) in comparison with other studies (men: 50.2 ± 5.2 SD ; women 46.9 ± 4.4 SD^9^^)^ and aged 40-49 years^10^^)^) and that green tea consumption is more common in elder than younger populations^25^^)^. Because major occupations of our subjects were farmers, self-employed and house-wives, it could lead to high intakes of green tea.

In conclusion, in this study of 12-day diet records collected from 119 men and women in rural northern Japan, we found rice and miso soup as important contributors of energy and macronutrients, and several characteristic dishes as the largest contributors of important nutrients. Our results also suggest that the examination of nutrient sources based on dishes and recipes, rather than on food materials, may be useful in characterizing the dietary patterns of populations. We used these data to develop a food frequency questionnaire (FFQ) for a rural Japanese population. Validation of the questionnaire is currently underway.

## References

[1] Block G, Dresser CH, Hartman AM, Carroll MD. Nutrient Sources in the American diet: quantitative data from the NHANES II survey. I. Vitamins and minerals. Am J Epidemiol, 1985; 122: 13-26. 4014190 10.1093/oxfordjournals.aje.a114072

[2] Block G, Dresser CH, Hartman AM, Carroll MD. Nutrient sources in the American diet: quantitative data from the NHANES II survey. II. Macronutrients and fats. Am J Epidemiol, 1985; 122: 27-40. 4014199 10.1093/oxfordjournals.aje.a114084

[3] Krebs-Smith SM, Cronin FJ, Haytowits DB, Cook DA. Food sources of energy, macronutrients, cholesterol, and fiber in diets of women. J Am Diet Assoc, 1992; 92: 168-174. 1310699

[4] Subar AF, Krebs-Smith SM, Cook A, Kahle LL. Dietary sources of nutrients among US adults, 1989 to 1991. J Am Diet Assoc, 1998; 98: 537-547. 9597026 10.1016/S0002-8223(98)00122-9

[5] Freudenheim JL, Krogh V, D’Amicis A, . Food sources of nutrients in the diet of elderly Italians: I. Macronutrients and lipids. Int J Epidemiol, 1993; 22: 855-868. 8282465 10.1093/ije/22.5.855

[6] Krogh V, Freudenheim JL, D’Amicis A, . Food sources of nutrients in the diet of elderly Italians: II. Micronutrients. Int J Epidemiol, 1993; 22: 869-877. 8282466 10.1093/ije/22.5.869

[7] Cade JE, Margetts BM. Nutrient sources in the English diet: quantitative data from three English towns. Int J Epidemiol, 1988; 17: 844-848. 3225094 10.1093/ije/17.4.844

[8] Winkler G, Doring A, Keil U. Trends in dietary sources of nutrients among middle-aged men in southern Germany. Results of the MONICA Project Augsburg: dietary surveys 1984/1985 and 1994/1995. Monitoring trends and determinants in cardiovascular diseases. Appetite, 2000; 34: 37-45. 10744890 10.1006/appe.1999.0273

[9] Tsubono Y, Takamori S, Kobayashi M, . A data-based approach for designing a semiquantitative food frequency questionnaire for a population-based prospective study in Japan. J Epidemiol, 1996; 6: 45-53. 8795957 10.2188/jea.6.45

[10] Tokudome S, Ikeda M, Tokudome Y, . Development of data-based semi-quantitative food frequency questionnaire for dietary studies in middle-aged Japanese. Jpn J Clin Oncol, 1998; 28: 679-687. 9861235 10.1093/jjco/28.11.679

[11] Tokudome Y, Imaeda M, Ikeda M, . Foods contributing to absolute intake and variance in intake of fat, fatty acids and cholesterol in middle-aged Japanese. J Epidemiol, 1999; 9: 78-90 10337080 10.2188/jea.9.78

[12] Imaeda N, Tokudome Y, Ikeda M, . Foods contributing to absolute intake and variance in intake of selected vitamins, minerals and dietary fiber in middle-aged Japanese. J Nutr Sci Vitaminol, 1999; 45: 519-532. 10683805 10.3177/jnsv.45.519

[13] Borrud LG, McPherson RS, Nichaman MZ, Pillow PC, Newell GR. Development of a food frequency instrument: ethnic differences in food sources. Nutr Cancer, 1989; 12: 201-211. 2771799 10.1080/01635588909514020

[14] Thompson FE, Sowers MF, Frongillo EA Jr, Parpia BJ. Sources of fiber and fat in diets of US women aged 19 to 50: implications for nutrition education and policy. Am J Public Health, 1992; 82: 695-702. 1314519 10.2105/ajph.82.5.695 PMC1694142

[15] Block G, Norris JC, Mandel RM, DiSogra C. Sources of energy and six nutrients in diets of low-income Hispanic-American women and their children: quantitative data from HHANES, 1982-1984. J Am Diet Assoc, 1995; 95: 195-208. 7852686 10.1016/S0002-8223(95)00048-8

[16] Hampl JS, Betts NM. Comparisons of dietary intake and sources of fat in low- and high-fat diets of 18- to 24-year-olds. J Am Diet Assoc, 1995; 95: 893-897. 7636080 10.1016/s0002-8223(95)00247-2

[17] Kuhnlein HV, Soueida R, Receveur O. Dietary nutrient profiles of Canadian Baffin Island Inuit differ by food source, season, and age. J Am Diet Assoc, 1996; 96: 155-162. 8557942 10.1016/S0002-8223(96)00045-4

[18] Ballew C, White LL, Strauss KF, Benson LJ, Mendlein JM. Intakes of nutrients and food sources of nutrients among the Navajo: findings from the Navajo Health and Nutrition Survey. J Nutr, 1997; 127(10 Suppl): 2085S-2093S. 9339174 10.1093/jn/127.10.2085S

[19] Fukao A, Tsubono Y, Komatsu S, . A cohort study on the relation of lifestyle, personality and biologic markers to cancer in Miyagi, Japan: study design, response rate and profiles of the cohort subjects. J Epidemiol, 1995; 5: 153-157.

[20] Tsuji I, Nishino Y, Ohkubo T, . A prospective cohort study on National Health Insurance beneficiaries in Ohsaki, Miyagi Prefecture, Japan: study design, profiles of the subjects and medical cost during the first year. J. Epidemiol, 1998; 8: 258-263. 9884474 10.2188/jea.8.258

[21] Science and Technology Agency. Standard tables of food composition in Japan. 4th revised ed. Printing Bureau, Ministry of Finance, Tokyo, 1982. (in Japanese)

[22] Adachi M. Jitsubutsudai Sonomanma Ryoricard. First ed. Gunyousya, Tokyo, 1994. (in Japanese)

[23] Block G, Hartman AM, Dresser CM, . A data-based approach to diet questionnaire design and testing. Am J Epidemiol, 1986; 124 : 453-469. 3740045 10.1093/oxfordjournals.aje.a114416

[24] Ogawa K, Tsubono Y, Nishino Y, . Inter- and intra-individual variation of food and nutrient consumption in a rural Japanese population. Eur J Clin Nutr, 1999; 53: 781-785. 10556984 10.1038/sj.ejcn.1600845

[25] Tsubono Y, Nishino Y, Komatsu S, . Green tea and the risk of gastric cancer in Japan. N Engl J Med, 2001; 344, 632-636. 11228277 10.1056/NEJM200103013440903

